# Notes of the Week

**Published:** 1921-04-30

**Authors:** 


### NOTES OF THE WEEK.

### A SAD DECISION.

The action of King's College Hospital in deciding to reduce the number of beds available for the use of the general public to two hundred is one which must be received with keen regret by all interested in this splendid hospital and in the voluntary system generally. The wonderful building at Denmark Hill, which is still the last word in hospital architecture, opened its doors under the best auspices. There was every hope that the move from Portugal Street would be in every way, financially and otherwise, successful. But the war, which has wrecked and changed so much, could not be foreseen, and what appeared to be prudent expenditure has so multiplied that any method of overcoming the difficulty, other than the method decided upon, seems for the moment hard to find. A debt of £80,000 at the end of 1920, which has been increased at the rate of £1,000 per week since, is terrifying indeed, and cannot be overtaken sufficiently quickly by the patients' payment scheme that has been instituted, or by the constant efforts to improve income that are being made in all directions. That there is a waiting-list of 1,000 patients is a fact that does not make the story more pleasant, but it is one which was doubtless carefully weighed by the committee when they arrived at their sad decision. Except to its patients and its district King's College Hospital is not too easy of access, and we are sure that if a hundred rich men could be taken to Denmark Hill to see the beautiful building and the excellent work that is done within, the present disaster would be averted.

### A CENTRE OF BRITISH MEDICINE.

We understand that a scheme is already being considered for an arrangement between the Fellowship of Medicine and the Great Northern Central Hospital, whereby advanced medical teaching may be brought to one centre. It is, without being in possession of full details, impossible to comment freely upon this plan and its possibilities, but, if gradual development of the ideal post-graduate teaching hospital is foreshadowed, then British medicine stands to gain greatly by any satisfactory arrangement arrived at.

### A MEDICAL KNIGHTHOOD.

The King has conferred a Knighthood on Dr. James Craig, F.R.C.P., who is the King's Professor of Medicine at Trinity College, Dublin, and President of the Royal College of Physicians of Ireland. He is also the physician-in-ordinary to the Lord-Lieutenant of Ireland.

### HOSPITALS AND INSURED PATIENTS.

Representatives of some of the larger Approved Societies and members of the Executive of the British Hospitals Association met under the chairmanship of Viscount Hambleden last week, and it was agreed that the British Hospitals Association should ascertain the views of hospitals throughout the country on several points as to the treatment of insured patients at voluntary hospitals. It is to be ascertained if hospitals are prepared to accept from the societies an agreed percentage of the cost of treating members as in- or out-patients; whether they are prepared to agree that a payment, direct or indirect, equivalent in value to such percentage should be required by hospitals in respect of treatment afforded to members of societies not so contributing; and whether, assuming equal urgency for in-patient treatment on the part of persons desiring the same, some measure of priority should be given to members of contributing societies. It is stated that if an affirmative response is given, if such questions could be answered by a simple affirmative, the parties will meet again to draft a definite scheme.

### PSYCHO-ANALYSIS.

We trust that our general readers will appreciate the severe comments recently made by a coroner on the circumstances of the suicide of a patient after her visit to a so-called psycho-analyst. One does not suggest that this particular instance throws much light on the merits or demerits of psycho-analysis in itself. But the point which it certainly does raise is that this fashion may carry with it an element of real danger, and that its practice should rapidly be brought under some definite regulations and restrictions.

### "A SLAVE TO THE HOSPITAL."

Mr. A. Bird has retired from the secretaryship of the Dudley Guest Hospital after twenty-seven years' work. In announcing this at the meeting of the Workmen's Committee, Mr. F. J. Ballard said that when he saw Mr. Bird the day after he had absented himself from the hospital, Mr. Bird said that he would never return to the institution, though he made no complaint. Mr. Ballard could explain this action only through a breakdown in health after Mr. Bird's recent operation, which had caused some of his work to fall into arrears. Mr. Bird had been a slave to the hospital, had raised the greater part of its income, and his services could hardly be over-estimated. A resolution of unabated confidence in Mr. Bird was passed and a deputation appointed to ask him to reconsider his decision. Meanwhile Mr. Raymond Hurst, who has given much assistance already, has been appointed acting secretary for three months.

### THE HOSPITAL POOL.

The medical correspondent of *The Times* very wisely says that the next few months promise to prove a testing time for the hospitals such as they have not hitherto experienced. If they surmount the crisis, he considers, their future should be more secure than it has been during many years. The first of the steps that will be suggested by Lord Cave's Committee, it is foreshadowed, will be concerted action as regards both raising funds and spending them; this action probably will be, in London, undertaken by King Edward's Hospital Fund, and in the provinces by committees appointed by the individual committees, and these bodies will act as intermediaries between Government and hospitals for the reception and distribution of grants in aid. It is a testimony to the respect which is generally accorded to the King's Fund that it could be possible to make the paradoxical suggestion that a self-appointed body should reign in London while a popularly elected body undertakes the same office in the rest of the country.

### DEATH OF MR. EDMUND FORSTER.

We regret to record the death of Mr. Edmund Forster, M.A., which took place a fortnight ago. On account of ill-health, due to the work and anxieties of the war, he resigned the post of superintendent and secretary to the Derbyshire Royal Infirmary in 1918, and since then lived at Castle Donington in Derbyshire, where he endeared himself to all classes. Mr. Forster, on leaving the University, entered the teaching profession, but relinquished that in favour of hospital work in 1899 at the age of thirty-four, when he became house governor and secretary to Wolverhampton General Hospital. In 1905 he was appointed secretary to the Bradford Royal Infirmary, and a year later was selected for the Derbyshire vacancy. He was the first President of the Midlands Branch of the Incorporated Association of Hospital Officers, and was liked and highly esteemed by his colleagues in the hospital world. He had many interests besides his work, and was a man of much culture and judgment.

### THE ROCHDALE INFIRMARY SECRETARY.

Mr. Walter Wynne, who has held the post of chief clerk in the Superintendent's office at the Salford Royal Hospital for the past two years, has been elected secretary to the Rochdale Infirmary. Prior to his appointment at Salford Mr. Wynne was chief clerk in the Secretary's office at the Chester Royal Infirmary.

### THE GOVERNMENT AND HOSPITAL RESEARCH.

The Royal Mineral Water Hospital, Bath, has acquired the adjoining building hitherto used as the Bath Blue-coat School. In connection with the treatment of rheumatism, which a number of rheumatic soldiers underwent during the war, research and experiments have been made. We understand that, in consequence of a recent visit paid by a specialist from the Ministry of Health, Government recognition might be extended if the research were extended. The recent extension will give opportunities for this, and it will be interesting to learn what form Government recognition will take.

### HEADACHE.

Medically, headache is generally considered as a relatively unimportant if annoying symptom. Its possible importance is, however, well brought out in the last issue of *The Times Trade Supplement*. The appeal is as much to employers of labour as to the workers themselves, and they may both well remember that a decided tendency to headache marks a distinct departure from normal health. The great point made is that persistent headache is one of the most obvious, and yet most often missed, danger-signals in general and industrial medicine. At the same time, if this indication be accepted with reasonable promptness, in the great majority of cases the trouble is still in a curable state. Admittedly, there are exceptions, but they are relatively few. From consideration of either health or efficiency, headache is of first importance to the employer of labour.

### CO-OPERATION IN MANCHESTER.

As already noted in The Hospital, a serious attempt is being made in Manchester and Salford to secure by combined effort a better result from employers' and employees' contributions. As long ago as last December a committee representing the thirteen principal institutions was formed, and a month later a sub-committee was formed to draft a scheme. But since the aim includes some system of joint publicity or appeal, it is desirable that the Manchester and Salford Hospital Saturday and Convalescent Fund should be included, if only that no accredited agency may be left out. For the same reason help may be expected from the Manchester and Salford Council of Social Service and from the Manchester and Salford Medical Charities Hospital Sunday Fund. The Salford Royal and Ancoats Hospitals have already a scheme of their own, and since this is a local effort it should help rather than hinder additional joint action. The line of least resistance is, however, that of increasing employers' and workers' contributions, partly because such a scheme directly benefits all hospitals and partly because all jointly gain indirectly from the public interest thus created. The individualism which is part of the voluntary system makes progress necessarily slow, but since goodwill is generally felt, this is not to be regretted, for co-operation has yet to find the best form of common action. The convalescent homes, for instance, have the first claim on the Saturday Fund, and probably the best way to preserve this precedence and to increase the grants made to hospitals is to enlarge the scope of the contributions so as to secure support for the hospitals on an equally generous scale.

### THE HOSPITAL FAMILY.

The Liverpool Council of Voluntary-Aid is doing, as we have noticed, useful work in many respects. Its publications, particularly the Hand-book, now in its third edition, gives the excuse and justification of the co-ordination which it is the Council's business to secure between the different charities. How numerous these are is a little staggering. The index of organisations alone runs to twenty pages of names, and includes hospitals, homes, police-court mission, employment agencies, and the like. It is very remarkable that voluntary hospitals, which were the first great institutions of this class, have grown in numbers far less repidly than these auxiliary bodies, which are in fact offshoots of the hospital spirit. It is this disparity of numbers which the hospitals have to make good; for as things are, not only is there a serious lack of beds, but the younger institutions are apt perhaps to think that all progress lies with them, and that the hospitals from which they originated are falling behind in the race.

